# A Multicenter, Open-Label Study of Combined Poly-L-Lactic Acid and Hyaluronic Midface Filler Regimen Enhances Facial Harmony and Skin Quality in GLP-1 Medication Users

**DOI:** 10.1093/asj/sjaf240

**Published:** 2025-11-17

**Authors:** Z Paul Lorenc, Michael Somenek, Thu Q Nguyen, Sindhu Garimella, Jessica Hicks, Jennifer H T D Le, Matthew H Meckfessel

## Abstract

**Background:**

Weight loss induced by glucagon-like peptide-1 receptor agonists (GLP-1 RAs) can lead to facial volume loss, wrinkles, and sagging skin, resulting in an aged and gaunt appearance that negatively affects subject satisfaction and self-perception.

**Objectives:**

This study assessed the treatment regimen of poly-L-lactic acid (PLLA-SCA) and 2 hyaluronic acid midface fillers (HA-LYF, HA-CON) on restoring facial balance, correcting contour deficiencies, and improving skin quality in subjects who experienced weight loss from GLP-1 RA therapy.

**Methods:**

This multicenter, open-label study enrolled 41 subjects with cheek wrinkles and midface contour deficiencies following GLP-1 RA–driven weight loss. Subjects received 2 to 3 treatment sessions of PLLA-SCA and 1 to 2 treatment sessions of HA-LYF or HA-CON, with follow-ups through 9 months since the last PLLA-SCA treatment. Efficacy evaluations included objective skin quality assessments using bioinstrumentation, improvements in facial contour deficiencies using standardized photography, and subject self-assessment questionnaires. Safety was also monitored throughout the study.

**Results:**

The PLLA-SCA and HA-LYF or HA-CON treatment regimen significantly improved facial skin quality and enhanced contour in the cheek, jawline, and perioral areas, and demonstrated objective improvement in hydration and skin radiance. Additionally, subjects were satisfied with the treatment results, with no treatment-related adverse events reported.

**Conclusions:**

PLLA-SCA and HA-LYF or HA-CON provided effective, safe, and sustained improvements in facial balance, contour, and skin quality in subjects who experienced facial volume loss and ageing from weight loss following GLP-1 RA therapy.

**Level of Evidence: 3 (Therapeutic):**

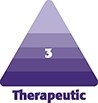

Obesity is a highly prevalent chronic condition associated with comorbidities that severely impact health and contribute to increased morbidity and mortality.^1,2^ Glucagon-like peptide-1 receptor agonist (GLP-1 RA) therapies have demonstrated considerable effectiveness in promoting sustained reductions in weight, body mass index (BMI), and waist circumference in clinical and real-world studies.^1,3^ Furthermore, GLP-1 RAs improve hyperglycemia and blood pressure, reduce inflammation, and provide cardiometabolic and renal protection.^1,4^

GLP-1 RA–driven weight loss can lead to changes throughout the body and cause unintended changes to the face, including wrinkles and sagging skin, which are particularly prominent in areas such as the temples, cheeks, tear troughs, jawline, marionette lines, and nasolabial folds.^5,6^ It may also cause changes to the size of the lips, cheeks, and chin due to the loss of collagen and elastin, disrupting the balance of facial features and resulting in a gaunt and aged facial appearance. Furthermore, GLP-1 RA–driven loss of collagen, elastin, and fatty acids affects the skin structure and barrier, leading to dryness and a lackluster appearance, effects that are particularly noticeable in older adults with reduced collagen and elastin levels in their skin.^5^ In contrast, reports of general rapid weight loss unrelated to GLP-1 RAs emphasize loose or sagging skin, more hollowed-out cheeks, sunken areas beneath the eyes, and more pronounced nasolabial folds.^7^

Facial fat loss is rarely reported as an undesired or adverse effect in clinical trials evaluating GLP-1 RAs for weight loss.^3,5^ Additionally, subjects are often unaware of the potential changes in facial appearance following rapid weight loss and the physical and emotional impact of these changes.^5^ Indeed, studies show that subjects who experience extensive weight loss (>100 lbs) appear up to 5.1 years older than those of a similar age without a history of overweight or obesity.^6-8^ The facial changes associated with minimal or significant medication-driven weight loss may lead patients to explore aesthetic improvements, supporting the integration of aesthetic management into GLP-1 RA–driven weight loss.^7,8^

Restoration of facial volume and correction of facial changes following GLP-1 RA–driven weight loss can be achieved through several aesthetic interventions, including the use of dermal fillers, skin-tightening techniques, and surgery.^5,6^ The hyaluronic acid (HA)-based dermal fillers, Restylane Lyft with Lidocaine (HA-LYF, Q-MED-AB, Galderma, Lausanne, Sweden) and Restylane Contour (HA-CON, Q-MED-AB, Galderma; also referred to as Restylane Volyme outside of the United States), have demonstrated efficacy in cheek augmentation and correction of contour deficiencies.^9-11^ In contrast to HA fillers, biostimulatory injectables, such as poly-L-lactic acid (Sculptra [PLLA-SCA], Q-MED-AB, Galderma), can restore facial volume and improve skin quality through biostimulation,^12-14^ and repeated PLLA-SCA treatments have been found to improve skin quality and volume through tissue remodeling.^15,16^

Despite the well-characterized clinical effects of HA-LYF, HA-CON, and PLLA-SCA, there is a lack of data to evaluate their effects in GLP-1 RA–driven weight loss and objectively assess associated improvements in skin quality. Bioinstrumentation enables objective quantification of skin quality changes. These tools include Corneometer for measuring skin hydration, Glossymeter for skin radiance, and ultrasound for skin thickness, allowing for detailed analysis of skin parameters to assess treatment efficacy.

With the rise of GLP-1 RA–driven weight loss, there is a need for evidence-based outcomes in facial injectable aesthetics. A treatment regimen of PLLA-SCA and HA-CON or HA-LYF filler injections could be an effective approach to achieving both long-lasting and immediate facial rejuvenation. This study assessed the effects of PLLA-SCA with HA-CON or HA-LYF for cheek augmentation and correction of contour deficiencies in subjects with GLP-1 RA–driven weight loss.

## METHODS

### Study Design

This multicenter, open-label study was conducted in the United States between February 2024 and May 2025 (clinicaltrials.gov number: NCT06351358). Written informed consent was obtained from all subjects before study participation, and the Institutional Review Board (IRB) approved the protocol on February 8, 2024 (IRB ID: 11677). The study was conducted in accordance with Good Clinical Practices, the ethical principles contained within the Declaration of Helsinki, and all applicable regulatory requirements.

**Figure 1.**
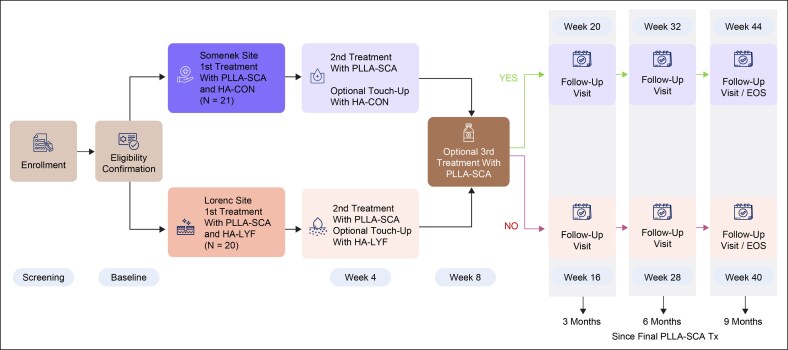
Study flow diagram. EOS, end of study; HA-CON, hyaluronic acid-Restylane Contour; HA-LYF, hyaluronic acid-Restylane Lyft with Lidocaine; PLLA-SCA, poly-L-lactic acid-Sculptra; Tx, treatment.

Eligible subjects (*N* = 41) received an initial treatment regimen of PLLA-SCA and HA-LYF or HA-CON at the baseline visit. At Week 4, subjects received a second PLLA-SCA treatment and an optional touch-up of HA-LYF or HA-CON for optimal correction. At Week 8, they received an optional PLLA-SCA treatment for optimal correction. Subjects had follow-up visits at 12-week intervals following the last PLLA-SCA treatment at Months 3, 6, and 9 (Figure 1).

### Study Population

The study enrolled adult females (*n* = 39) and males (*n* = 2), aged 22 years or older, of any race, ethnicity, and Fitzpatrick skin type (FST), with moderate-to-severe cheek wrinkles and mild-to-severe midface contour deficiencies. Subjects were included if they were currently taking or had a history of taking GLP-1 RA treatment, maintained a stable BMI within 4 to 6 weeks before the study start, and were willing to maintain their BMI (±2 kg/m^2^) throughout the study. Subjects were ineligible to participate in the study if they had received treatment with collagen or HA within the past 12 months, received calcium hydroxyapatite, PLLA, or fat within the past 36 months, or who had a history of injectable polymethylmethacrylate treatment. Subjects with contraindications to injections or allergy or hypersensitivity to any ingredient in the treatment products were also excluded.

### Study Treatment

PLLA-SCA (Sculptra, Q-MED-AB, Galderma) was reconstituted in 8 mL sterile water with 1 mL of optional 2% lidocaine and injected into the lower third of the face and the pyriform fossa in the supraperiosteal and subcutaneous regions using a fanning injection technique with a 25-gauge needle (Supplemental Figure 1). PLLA-SCA was administered at baseline (Day 0) and at Week 4, with an optional dosing at Week 8.

HA-LYF or HA-CON (Restylane Lyft with Lidocaine, Restylane Contour [United States]/Restylane-Volyme [rest of world], Q-MED-AB, Galderma) were injected into the zygoma in the supraperiosteal or subcutaneous regions using a fanning injection technique with a 25-gauge cannula (Supplemental Figure 1). HA-LYF or HA-CON was administered at baseline (Day 0), with an optional dosing of either HA-LYF or HA-CON at Week 4.

Subjects attended 3 follow-up visits at Months 3 (±7 days), 6 (±7 days), and 9 (±7 days) after the last PLLA-SCA injection.

### Study Assessments

The primary objective was to assess the efficacy of treatments on skin improvement using bioinstrumentation and clinical photography at each follow-up visit compared to baseline. The secondary objective was to evaluate subject satisfaction with their cheeks using self-assessment questionnaires after treatment. The safety objective evaluated adverse events (AEs) and device deficiencies throughout the study.

Efficacy was assessed through clinical photography, bioinstrumentation measurements, and self-assessment questionnaires (Appendix). Clinical photography of each subject's face (left, front, and right views) was performed using the VISIA-CR photo station (Canfield Imaging Systems, Parsippany, NJ) with a digital SLR camera under standard, cross-polarized, and parallel-polarized lighting conditions.

Bioinstrumentation measurements were performed (at 1 study site only) on each subject's right side using Corneometer (Courage + Khazaka, Köln, Germany) to assess skin hydration, Glossymeter (Courage + Khazaka) to assess skin radiance, and DermaLab ultrasound (Cortex Technology, Aalborg, Denmark) to assess skin thickness. Measurements were taken in the zygoma area of the HA-LYF–treated region (extending downward from the corner of the eye) and in the lower third of the face of the PLLA-SCA–injected region (2 to 3 cm laterally from the end of the earlobe).

The self-assessment questionnaire asked subjects about their perception and satisfaction with the study treatments at each visit after baseline. The questionnaire was completed online at the study site, and all responses were collected and securely stored anonymously.

AEs were assessed at each visit through evaluations and interviews with subjects, and close monitoring by physicians. Subjects also maintained a daily diary to record any reactions or AEs in the treated areas.

### Statistical Analysis

All endpoints were summarized descriptively for the observed value as well as change from baseline. Bioinstrumentation data (skin hydration, skin radiance, and skin thickness) were analyzed using a one-sample t-test. If normality was not achieved, a Wilcoxon signed-rank test was used. For bioinstrumentation data, an increase in scores indicates an improvement. The self-assessment questionnaire was tabulated for frequency and percentage of subject perception in which favorable responses were calculated using binomial tests. All quantitative data were presented as mean ± standard deviation. Categorical data were presented as frequencies or percentages, with *P* < .05 considered statistically significant. Due to subject dropout at the site assessing bioinstrumentation, a last observation carried forward was utilized to ensure statistical power throughout the study.^17^

## RESULTS

### Study Population

Among the 41 subjects who were enrolled in the study, the majority were female (95.1%; *n* = 39), White/Caucasian (70.7%; *n* = 29), and presented with FSTs II (36.6%; *n* = 15) and III (36.6%; *n* = 15), with a mean age of 58.9 years (range, 32-79 years; Table 1). Six subjects discontinued the study due to loss to follow-up (*n* = 3), voluntary withdrawal (*n* = 2), and noncompliance (*n* = 1). The majority of subjects attended their follow-up visits as scheduled. However, 26 subjects had follow-up deviations due to scheduling issues. These follow-up deviations ranged from 1 to 84 days (mean, 12.5 days), with the single 84-day deviation corresponding to the Month 9 follow-up visit at the HA-CON study site.

**Table 1.** Subject Demographics

Subject numbers		*N*	%
Enrolled		41	
Completed		35^a^	
Age	Mean	58.9	
	Minimum	32	
	Maximum	79	
Gender	Female	39	95.1
	Male	2	4.9
Race/Ethnic background	White/Caucasian	29	70.7
	Black/African American	3	7.3
	Hispanic/Latin American	6	14.6
	South Asian	1	2.4
	Southeast Asian	1	2.4
	Other	1	2.4
Fitzpatrick skin type	I	4	9.8
	II	15	36.6
	III	15	36.6
	IV	2	4.9
	V	5	12.2
	VI	0	0
Prior GLP-1 RA usage	Ozempic	15	36.6
	Mounjaro	8	19.5
	Wegovy	5	12.2
	Compounded Semaglutide	13	31.7

GLP-1, glucagon-like peptide-1; RA, receptor agonist.

^a^Discontinuations were due to lost to follow-up (*n* = 3), noncompliance (*n* = 1), and requested withdrawal (*n* = 2). None were due to adverse events.

GLP-1 RA usage in subjects prior to enrolling in the study included Ozempic (*n* = 15), Mounjaro (*n* = 8), Wegovy (*n* = 5), and compounded semaglutide (*n* = 13), and the mean duration of GLP-1 RA usage was 1.69 years (Table 1). All but one subject was on GLP-1 medication for the duration of the study. Mean weight and BMI remained stable from baseline (mean weight, 154.68 lbs; mean BMI, 25.80; *n* = 41) to study completion (mean weight,152.69 lbs; mean BMI, 25.45; *n* = 35). Mean weight loss was 34.1 lbs (6 points BMI change) at the start of study.

At baseline, subjects received a mean injection dosage of 1.96 mL HA-LYF (*n* = 20) or 2.06 mL HA-CON (*n* = 21). At Week 4, dosing averaged 1.16 mL HA-LYF (*n* = 12) or 1.78 mL HA-CON (*n* = 18; Table 2). Mean PLLA-SCA dosages were 17.65 mL (*n* = 41) at baseline, 17.74 mL (*n* = 40) at Week 4, and 16.96 mL (*n* = 35) at Week 8 (Table 2).

**Table 2.** Study Treatments

			HA-LYF	HA-CON	PLLA-SCA
Baseline	Injected amount (mL)	N	20	21	41
		Minimum	1.6 mL	1 mL	14 mL
		Maximum	2 mL	4 mL	18 mL
		Average	1.96 mL	2.06 mL	17.65 mL
Week 4	Injected amount (mL)	N	12	18	40
		Minimum	0.5 mL	0.55 mL	14 mL
		Maximum	2 mL	3.1 mL	18 mL
		Average	1.61 mL	1.78 mL	17.74 mL
Week 8	Injected amount (mL)	N			35
		Minimum			9 mL
		Maximum			18 mL
		Average			16.96 mL

HA-CON, hyaluronic acid-Restylane Contour; HA-LYF, hyaluronic acid-Restylane Lyft with Lidocaine; PLLA-SCA, poly-L-lactic acid-Sculptra.

### Clinical Improvements in Facial Definition and Contour

**Figure 2.**
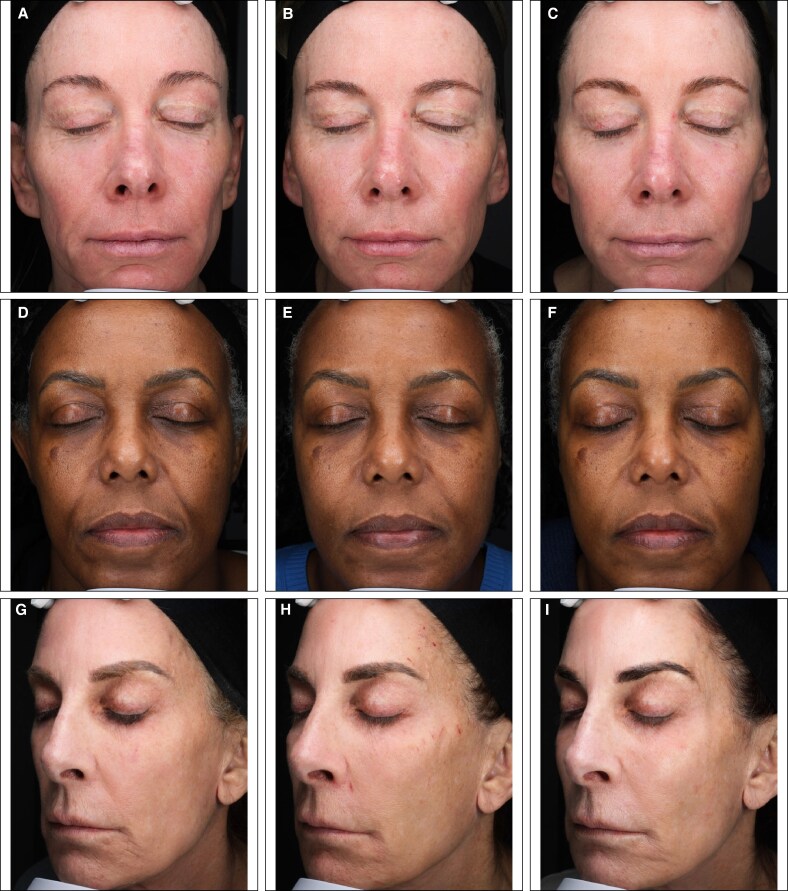

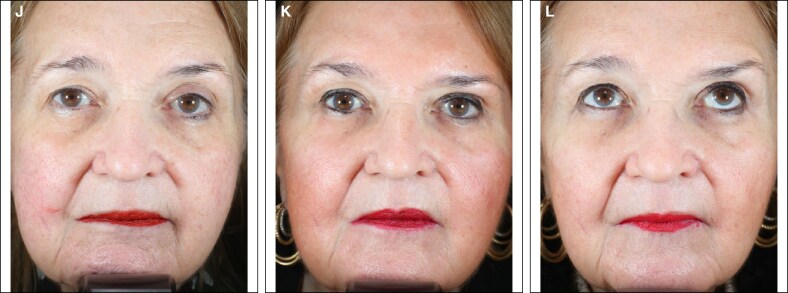
Clinical photography of changes in facial definition and contour. Subject 102, female, aged 59 years, White/Caucasian, Fitzpatrick skin type 2, standard light; treatment regimen (poly-L-lactic acid-Sculptra, 54 mL; hyaluronic acid-Restylane Contour, 4 mL; glucagon-like peptide-1 [Wegovy, 1 mg/weekly, 9.5 years]); weight loss, 47 lbs; at (A) baseline, (B) Month 3*, and (C) Month 9*. Subject 103, female, aged 59 years, Black/African American, Fitzpatrick skin type 5, standard light; treatment regimen (poly-L-lactic acid-Sculptra, 51.25 mL; hyaluronic acid-Restylane Contour, 3.70 mL; glucagon-like peptide-1 [Wegovy, 1 mg/weekly, 2 years (ongoing)]); weight loss, 43 lbs; at (D) baseline, (E) Month 3*, and (F) Month 9*. Subject 106, female, aged 62 years, White/Caucasian, Fitzpatrick skin type 2, standard light; treatment regimen (poly-L-lactic acid-Sculptra, 53 mL; hyaluronic acid-Restylane Contour, 4 mL; glucagon-like peptide-1 [semaglutide, 8-100 mg/biweekly, 3 years (ongoing)]); weight loss, 25 lbs; at (G) baseline, (H) Month 3*, and (I) Month 9*. Reproduced from Nikolis A, Enright KM, Fabi SG, et al, *Consensus Statements on Managing Aesthetic Needs in Prescription Medication–Driven Weight Loss Patients: An International, Multidisciplinary Delphi Study*, *Journal of Cosmetic Dermatology*, 2025, under the terms of the Creative Commons Attribution 3.0 License (CC BY 3.0). License available at https://creativecommons.org/licenses/by/3.0/. Subject 004, female, aged 72 years, Hispanic/Latin, Fitzpatrick skin type 3, standard light; treatment regimen (poly-L-lactic acid-Sculptra, 45.0 mL; hyaluronic acid-Restylane Lyft with Lidocaine, 3.1 mL; glucagon-like peptide-1 [Ozempic 2 mg/weekly, 2 years (ongoing)]); weight loss, 32 lbs; at (J) baseline, (K) Month 3*, and (L) Month 9*. *Indicates time since last PLLA-SCA treatment.

HA-LYF or HA-CON were injected into the zygoma to achieve immediate cheek augmentation and correction of contour deficiencies, while PLLA-SCA was injected into the lower face for volume restoration and collagen production, resulting in a natural appearance over time. The treatment regimen of PLLA-SCA and HA-LYF or HA-CON enhanced the aesthetic appearance in subjects experiencing weight loss with associated facial volume loss following GLP-1 RA treatments. Clinical photography demonstrated improvements in facial definition and contour from baseline to 9 months after PLLA-SCA injection (Figure 2). Notably, visible changes were observed in the cheek, jawline, and perioral areas (Figure 2). Furthermore, these benefits were maintained post-treatment through Month 9 (Figure 2).

### Hyaluronic Acid Significantly Improved Skin Hydration

**Figure 3.**
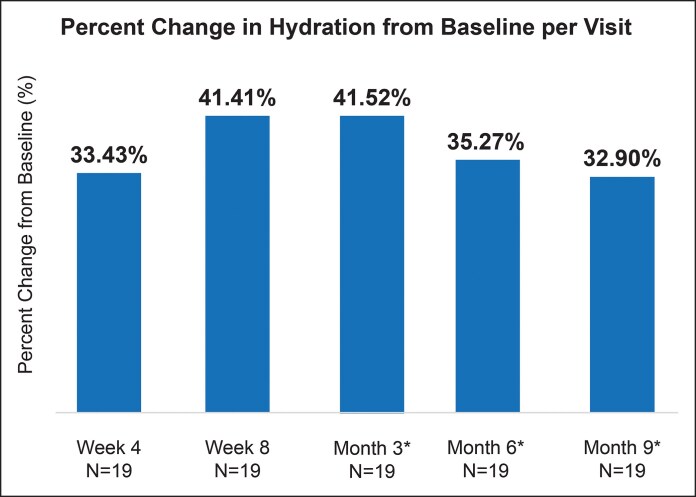
Skin hydration measured using Corneometer. Percent change in skin hydration from baseline per visit for hyaluronic acid-Restylane Lyft with Lidocaine. *Indicates time since last PLLA-SCA treatment.

The combined PLLA-SCA and HA-LYF regimen improved skin quality beyond volumization. Bioinstrumentation measurements showed sustained skin hydration benefits of HA-LYF, with significant improvements beginning at Week 4 (33.43% change from baseline; *P* = .0483) to Month 9 (32.90% change from baseline; *P* = .0227; Figure 3). However, skin hydration was not significantly changed with PLLA-SCA treatment (Supplemental Figure 2).

### Poly-L-Lactic Acid Significantly Improved Skin Radiance and Thickness

**Figure 4.**
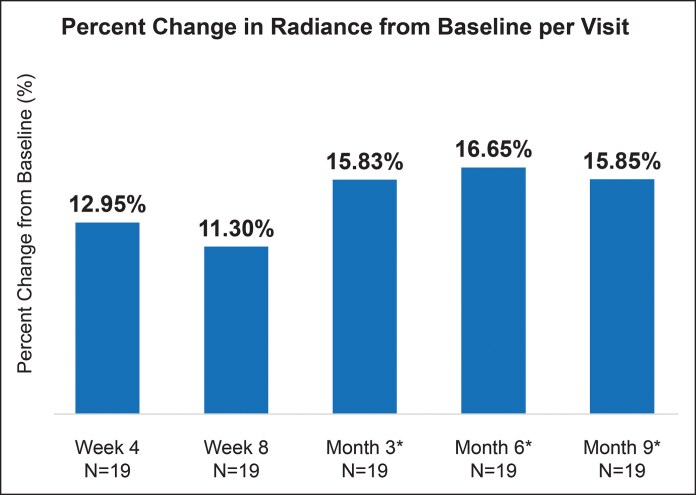
Skin radiance measured using Glossymeter. Percent change in skin radiance from baseline per visit for poly-L-lactic acid-Sculptra. *Indicates time since last PLLA-SCA treatment.

**Figure 5.**
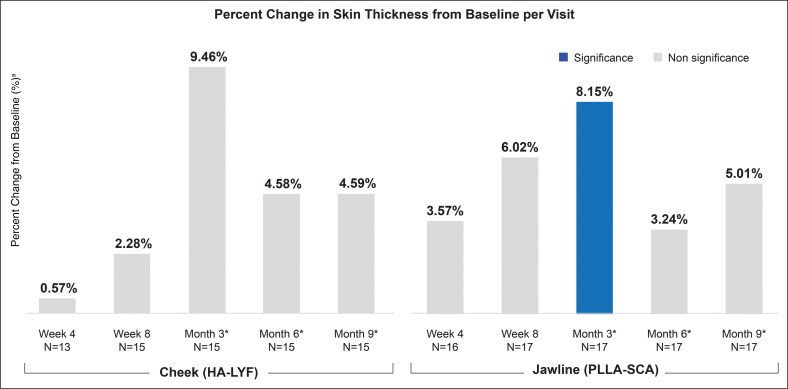
Skin thickness measured using ultrasound. Percent change in skin thickness from baseline per visit, stratified by treatment. ^a^Ultrasound data inconsistency may be due to probe sensitivity to pressure and location. As a result, some data points were not captured at each visit, as seen by the lower number of subjects, impacting statistical significance. HA-LYF, hyaluronic acid-Restylane Lyft with Lidocaine; PLLA-SCA, poly-L-lactic acid-Sculptra. *Indicates time since last PLLA-SCA treatment.

The PLLA-SCA treatment significantly improved skin radiance and skin thickness at Month 3. For skin radiance, bioinstrumentation measurements showed sustained effects of PLLA-SCA beginning at Week 4 (12.95% change from baseline; *P* = .0220), with more pronounced improvements at Month 3 (15.83% change from baseline; *P* = .0037) and Month 9 (15.85% change from baseline; *P* = .0122; Figure 4). However, treatment with HA-LYF along the zygoma decreased skin radiance from Months 3 to 9 (Supplemental Figure 3). Additionally, skin thickness was significantly increased at Month 3 (8.15% change from baseline; *P* < .05) and increased at Months 6 (3.24% change from baseline) and 9 (5.01% change from baseline; Figure 5).

### Subject-Reported Outcomes and Satisfaction

**Figure 6.**
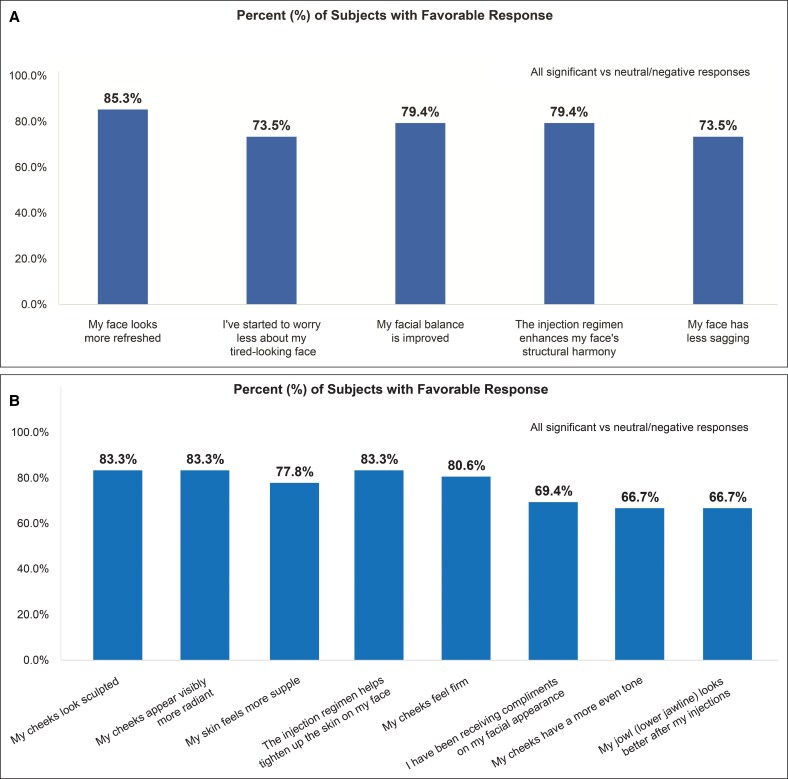
Subject satisfaction during the injection treatment period. Percentage of subjects with a favorable response at (A) Week 4 and (B) Week 8.

**Figure 7.**
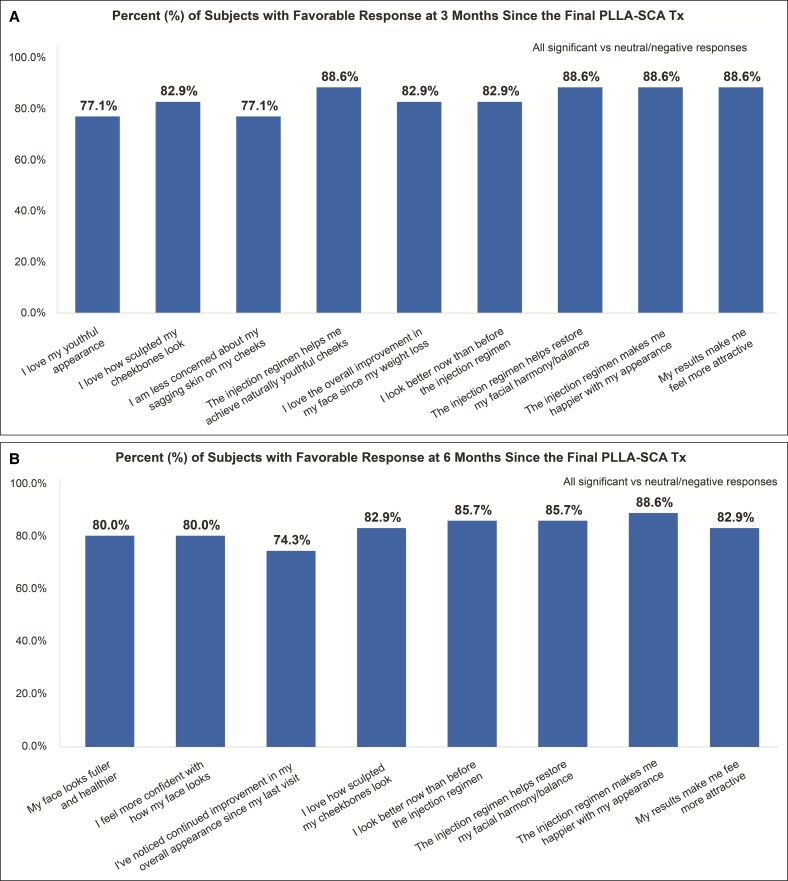
Subject satisfaction after injection treatments were completed. Percentage of subjects with a favorable response at (A) Month 3 and (B) Month 6 since the final poly-L-lactic acid-Sculptra treatment. PLLA-SCA, poly-L-lactic acid-Sculptra; Tx, treatment.

**Figure 8.**
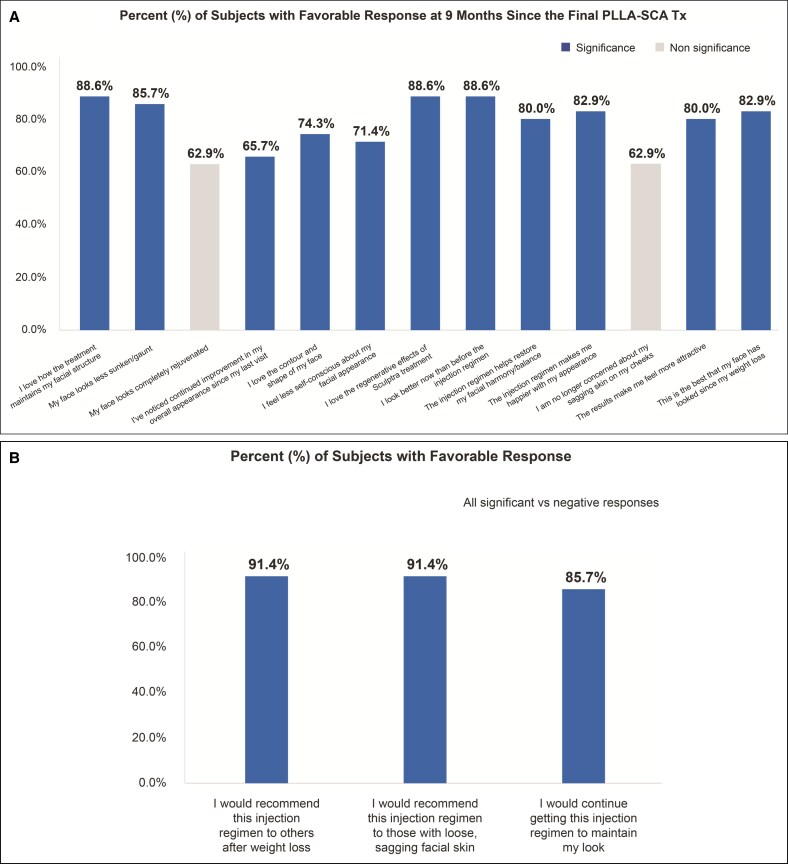
Subject (A) satisfaction and (B) recommendations at the end of the study. Percentage of subjects with a favorable response at Month 9 since the final poly-L-lactic acid-Sculptra treatment. PLLA-SCA, poly-L-lactic acid-Sculptra; Tx, treatment.

Subjects reported high satisfaction with treatment as early as Week 4 (Figure 6), which was maintained through Month 9 of the follow-up period (Figures 7, 8). Most subjects responded favorably to the satisfaction questionnaire, with >70% answering “agree” or “strongly agree” to 10 out of 13 questions (Figure 8A). At Month 9, 88.6% of treated subjects reported improved appearance compared to before the injection regimen (Figure 8A), and 91.4% would recommend the injection regimen to others after weight loss and to those with loose, sagging facial skin (Figure 8B).

By the end of the study, 88.6% of treated subjects expressed satisfaction with the regenerative effects of the PLLA-SCA treatment (Figure 8A). Additionally, most subjects (85.7%) expressed that their faces appeared less sunken and gaunt, and 82.9% reported that their faces looked the best since losing weight (Figure 8A).

Notably, a greater proportion of subjects expressed that the combined PLLA-SCA and HA-LYF/HA-CON regimen reduced their perceived age at the end of the study. Prior to the study, 60.0% of subjects felt they looked younger than their actual age, whereas at the end of the study, 82.9% of subjects felt they looked younger than their age (Supplemental Figure 4).

### Safety

The PLLA-SCA, HA-LYF, and HA-CON treatments exhibited a favorable safety profile, with no treatment-related AEs reported. Anticipated treatment-related reactions were mild and temporary, with 17 reports by subjects of minimal bruising, swelling, pain, bumps, redness, or soreness. These safety outcomes are consistent with previous safety data for all products.^9,11,14^

## DISCUSSION

While the skin quality benefits associated with PLLA-SCA injections are well established, this study is the first to demonstrate the skin quality benefits of improved hydration with HA-LYF injections into the zygoma. PLLA-SCA injections enhanced skin quality by regenerating collagen and elastin, leading to improved skin thickness at 3 months, radiance, and gradual wrinkle reduction.^12,13,15,18^ Additionally, PLLA-SCA helped restore skin volume and even out skin tone.^13,14^ Furthermore, this study shows that PLLA-SCA injections significantly improved skin radiance by bioinstrumentation, a finding supported by subject-reported improvements in skin radiance and overall facial appearance, which are consistent with reports from a previous study.^14^ However, ultrasound measurement for PLLA-SCA treatment did not reach significance at Months 6 and 9. Dermis echogenicity is influenced by several factors, including collagen fiber orientation, ground substance type, and water content.^19^

Weight loss resulting from GLP-1 RA therapy can lead to facial wrinkles, loss of firmness, and contour deficiencies, contributing to perceived ageing and dissatisfaction with facial appearance, which can have an emotional impact.^5-7^ The cheeks and jawline significantly contribute to overall facial aesthetics, with features such as an enhanced jawline.^20^ Studies have reported correlations between subject well-being and satisfaction with their appearance, and nonsurgical facial aesthetic treatments have demonstrated benefits in self-confidence, well-being, and quality of life.^20,21^ Dermal biostimulators (PLLA-SCA) and fillers (HA-LYF, HA-CON) provide nonsurgical therapeutic approaches to enhance facial aesthetics through cheek restructuring,^9,11,14^ with secondary effects that may improve jawline definition. HA-LYF is a firmer gel with a higher lifting capacity (G’) and targeted product integration, resulting in more projected results; in comparison, HA-CON is a more flexible gel with medium G′ and distributed product integration, leading to more contoured results.^22,23^ While high G′ gels like HY-LYF offer greater projection, fillers with lower G′, such as HA-CON, offer more flexibility to support dynamic expressions.^23,24^ These differences enable a more individualized approach to cheek augmentation and contour correction, tailored to a subject's facial characteristics, deficiencies, and aesthetic desires.^8,24^ This study demonstrated that the treatment regimen of PLLA-SCA and HA-LYF or HA-CON improved the subjects' satisfaction with their facial aesthetic, helping them to feel more attractive and less self-conscious.

Alongside rapid body fat reduction,^5^ GLP-1 RA therapy also depletes facial fat and volume^5^ and inhibits adipose stem cell differentiation and proliferation, as well as lipid accumulation in these cells, likely contributing to the observed weight loss effect.^25^ In addition to their benefits for weight loss and diabetes mellitus, GLP-1 RAs have effective anti-inflammatory properties that could contribute to improved skin quality.^4,26^ These anti-inflammatory effects occur directly, through interactions with GLP-1 receptors on immune cells, and indirectly, by improving glycemic control and promoting weight loss.^4,26^ Specifically, GLP-1 RAs lower HbA1c, inhibit the production of pro-inflammatory cytokines, and protect cardiovascular, neuronal, and renal cells by decreasing inflammation and apoptosis.^4,26^ However, it has been previously reported that the mode of action of PLLA-SCA involves inducing a localized subclinical inflammatory response at the injection site, which recruits neutrophils and macrophages, followed by fibroblasts, stimulating collagen production over time as the inflammation gradually reduces.^27^ Recent studies have also demonstrated that PLLA-SCA affected skin tissue layers and superficial and deep fat to promote the upregulation of gene expressions involved in the regeneration of endogenous collagen, elastin, and potentially adipocytes, while also inducing minimal, but essential, inflammation required for clinically meaningful regeneration.^15,18^ Notably, PLLA-SCA upregulates *ELN* (encodes tropoelastin, the precursor to elastin) and *MT1A* (encodes metallothionein-1A, a protective antioxidant).^15^ Despite the known anti-inflammatory effects of GLP-1 RAs, it did not appear to impact the efficacy of PLLA-SCA in this study, indicating its ability to act as a regenerative biostimulator in this population.

This study showed that PLLA-SCA improved skin radiance, but not hydration. Gene analysis revealed that PLLA-SCA upregulated *DKK1*, a regulator of skin pigmentation and thickness, by inhibiting WNT/beta-catenin signaling.^15^ These findings suggest that skin radiance could be influenced by other factors beyond hydration, such as more evened skin tone through pigmentation changes or improved organization of the stratum corneum, providing a possible explanation for the effects of PLLA-SCA on skin radiance previously observed.^14^ Further research is warranted to explore other biological factors influencing skin radiance and the broader effects of PLLA-SCA on these pathways.

Measuring subject perception is a key assessment because the goal of aesthetic procedures is often to improve appearance and well-being, which objective instrumentation may not fully capture. However, subject scores rely on individual interpretations of perceived improvements, making them subjective and prone to bias. This study objectively evaluated facial aesthetic procedures using measurable bioinstrumentation metrics to provide evidence-based data that support and corroborate more subjective satisfaction scores. This combined objective and subjective methodology for measuring the efficacy of PLLA-SCA, HA-LYF, and HA-CON injections provides a more comprehensive assessment of changes in cheek and jawline profiles and subject satisfaction in the GLP-1–treated population.

This study had the following limitations. It was an open-label and nonrandomized study, which may make it challenging to infer causality. Most of the enrolled subjects were female and had previously achieved their weight loss goals. This was to minimize the confounding effects that weight fluctuations may have on various outcome measures. Consequently, the findings may not be generalizable to males or individuals who are beginning their weight loss journey. However, most aesthetic treatment-seeking patients are female, and the study demographic likely reflects the real-world population. Additionally, the lack of a control group prevented distinction between the effects attributable to the study treatments and other unidentified factors. The 9-month study period limited the assessment of the long-term effects beyond this timeframe. However, other clinical studies have shown that both HA-LYF and HA-CON effectively corrected midface fullness and contour deficiencies, providing a high degree of aesthetic improvement and subject satisfaction, with benefits maintained for up to 12 or 18 months.^9-11^ Additionally, PLLA-SCA injections have been shown to significantly increase collagen type I while decreasing elastin fragmentation and wrinkle severity, as well as improving facial contours and skin quality (erythema, pore size, and roughness) compared to placebo, with clinical benefits maintained for up to 2 years.^12,14,18^ Bioinstrumentation measurements were captured for PLLA-SCA and HA-LYF but not HA-CON. However, since HA-LYF and HA-CON are both HA-based gels and produce comparable subject satisfaction outcomes, the HA-LYF bioinstrumentation results could likely extend to HA-CON. It is also notable that bioinstrumentation measurements, particularly ultrasound measurements for skin thickness, where precise probe location can be difficult to achieve during follow-up visits, require precise location and pressure. In addition, follow-up assessments of skin radiance and hydration may be affected by environmental conditions, such as colder seasons, which can reduce skin moisture, increase dryness, and induce cutaneous vasoconstriction in the cheeks following short-term cold exposure.^28^ Furthermore, the ultrasound measurements were based on a smaller subject group, yet an overall trend toward improvement was shown.

## CONCLUSIONS

This was the first study to investigate the treatment regimen of PLLA-SCA and HA-LYF or HA-CON in patients currently using GLP-1 RA medications who experienced medication-driven weight loss and subsequent changes in facial volume and structure. It was also the first study to objectively assess bioinstrumentation changes after treatment with PLLA-SCA and HA-LYF. The treatment regimen improved facial volume, contour deficiencies, and skin quality in this unique, yet growing, population. Treatments significantly enhanced skin hydration, radiance, and thickness, while providing both immediate and long-lasting improvements in facial definition and reducing signs of ageing and gauntness. High levels of subject satisfaction were observed throughout the study. Most subjects observed sculpting and regenerative treatment effects, reported they looked better post-treatment, and noted they would recommend the regimen to others. The study treatments exhibited a favorable safety profile, with no treatment-related AEs reported. These findings support the use of PLLA-SCA combined with HA fillers as a safe and effective nonsurgical approach for facial rejuvenation in subjects experiencing GLP-1 RA–induced weight loss.

## Supplemental Material

This article contains supplemental material located online at https://doi.org/10.1093/asj/sjaf240.

## Supplementary Material

sjaf240_Supplementary_Data
