# Supplementary material for: A Multicenter, Open-Label Study of Combined Poly-L-Lactic Acid and Hyaluronic Midface Filler Regimen Enhances Facial Harmony and Skin Quality in GLP-1 Medication Users
Source: Aesthet Surg J. 2025 Nov 17;46(5):509–19. doi: 10.1093/asj/sjaf240 (PMC13064655; doi:10.1093/asj/sjaf240)
Supplement: sjaf240_Supplementary_Data [file sjaf240_supplementary_data.zip › Appendix.docx]

**SELF-ASSESSMENT QUESTIONNAIRE**

**Week 4**

| *Thinking about how you looked before the study treatment, please answer the following questions* | Strongly agree | Agree | Neither agree nor disagree | Disagree | Strongly disagree |
| --- | --- | --- | --- | --- | --- |
| My face looks more refreshed | **🞏** | **🞏** | **🞏** | **🞏** | **🞏** |
| I’ve started to worry less about my tired-looking face | **🞏** | **🞏** | **🞏** | **🞏** | **🞏** |
| My facial balance is improved | **🞏** | **🞏** | **🞏** | **🞏** | **🞏** |
| The injection regimen enhances my face’s structural harmony | **🞏** | **🞏** | **🞏** | **🞏** | **🞏** |
| My face has less sagging | **🞏** | **🞏** | **🞏** | **🞏** | **🞏** |

**Week 8**

| *Thinking about how you looked before the study treatment, please answer the following questions* | Strongly agree | Agree | Neither agree nor disagree | Disagree | Strongly disagree |
| --- | --- | --- | --- | --- | --- |
| My cheeks look sculpted | **🞏** | **🞏** | **🞏** | **🞏** | **🞏** |
| My cheeks appear visibly more radiant | **🞏** | **🞏** | **🞏** | **🞏** | **🞏** |
| My skin feels more supple | **🞏** | **🞏** | **🞏** | **🞏** | **🞏** |
| The injection regimen helps tighten up the skin on my face | **🞏** | **🞏** | **🞏** | **🞏** | **🞏** |
| My cheeks feel firm | **🞏** | **🞏** | **🞏** | **🞏** | **🞏** |
| I have been receiving compliments on my facial appearance | **🞏** | **🞏** | **🞏** | **🞏** | **🞏** |
| My cheeks have a more even tone | **🞏** | **🞏** | **🞏** | **🞏** | **🞏** |
| My jowl (lower jawline) looks better after my injections | **🞏** | **🞏** | **🞏** | **🞏** | **🞏** |

**Week 16 or Week 20**

| *Thinking about how you looked before the study treatment, please answer the following questions* | Strongly agree | Agree | Neither agree nor disagree | Disagree | Strongly disagree |
| --- | --- | --- | --- | --- | --- |
| I love my youthful appearance | **🞏** | **🞏** | **🞏** | **🞏** | **🞏** |
| I love how sculpted my cheek bones look | **🞏** | **🞏** | **🞏** | **🞏** | **🞏** |
| I am less concerned about my sagging skin on my cheeks | **🞏** | **🞏** | **🞏** | **🞏** | **🞏** |
| The injection regimen helps me achieve naturally youthful cheeks | **🞏** | **🞏** | **🞏** | **🞏** | **🞏** |
| I love the overall improvement in my face since my weight loss | **🞏** | **🞏** | **🞏** | **🞏** | **🞏** |
| I look better now than before the injection regimen | **🞏** | **🞏** | **🞏** | **🞏** | **🞏** |
| The injection regimen helps restore my facial harmony/balance | **🞏** | **🞏** | **🞏** | **🞏** | **🞏** |
| The injection regimen makes me happier with my appearance | **🞏** | **🞏** | **🞏** | **🞏** | **🞏** |
| My results make me feel more attractive | **🞏** | **🞏** | **🞏** | **🞏** | **🞏** |

**Week 28 or Week 32**

| *Thinking about how you looked before the study treatment, please answer the following questions* | Strongly agree | Agree | Neither agree nor disagree | Disagree | Strongly disagree |
| --- | --- | --- | --- | --- | --- |
| My face looks fuller and healthier. | **🞏** | **🞏** | **🞏** | **🞏** | **🞏** |
| I feel more confident with how my face looks. | **🞏** | **🞏** | **🞏** | **🞏** | **🞏** |
| I've noticed continued improvement in my overall appearance since my last visit | **🞏** | **🞏** | **🞏** | **🞏** | **🞏** |
| I love how sculpted my cheek bones look | **🞏** | **🞏** | **🞏** | **🞏** | **🞏** |
| I look better now than before the injection regimen | **🞏** | **🞏** | **🞏** | **🞏** | **🞏** |
| The injection regimen helps restore my facial harmony/balance | **🞏** | **🞏** | **🞏** | **🞏** | **🞏** |
| The injection regimen makes me happier with my appearance | **🞏** | **🞏** | **🞏** | **🞏** | **🞏** |
| My results make me feel more attractive | **🞏** | **🞏** | **🞏** | **🞏** | **🞏** |

**Week 40 or Week 44**

| *Thinking about how you looked before the study treatment, please answer the following questions* | I look my age | 1-3 years younger | 4-6 years younger | 7-9 years younger | 10 or more years younger |
| --- | --- | --- | --- | --- | --- |
| How old do you think you looked before the study treatment injections? | **🞏** | **🞏** | **🞏** | **🞏** | **🞏** |
| How old do you think you look now compared to before the study treatment injections? | **🞏** | **🞏** | **🞏** | **🞏** | **🞏** |

| *Thinking about how you looked before the study treatment, please answer the following questions* | Strongly agree | Agree | Neither agree nor disagree | Disagree | Strongly disagree |
| --- | --- | --- | --- | --- | --- |
| I love how the treatment maintains my facial structure. | **🞏** | **🞏** | **🞏** | **🞏** | **🞏** |
| My face looks less sunken/gaunt. | **🞏** | **🞏** | **🞏** | **🞏** | **🞏** |
| My face looks completely rejuvenated. | **🞏** | **🞏** | **🞏** | **🞏** | **🞏** |
| I've noticed continued improvement in my overall appearance since my last visit | **🞏** | **🞏** | **🞏** | **🞏** | **🞏** |
| I love the contour and shape of my face | **🞏** | **🞏** | **🞏** | **🞏** | **🞏** |
| I feel less self-conscious about my facial appearance | **🞏** | **🞏** | **🞏** | **🞏** | **🞏** |
| I love the regenerative effects of Sculptra treatment | **🞏** | **🞏** | **🞏** | **🞏** | **🞏** |
| I look better now than before the injection regimen | **🞏** | **🞏** | **🞏** | **🞏** | **🞏** |
| The injection regimen helps restore my facial harmony/balance | **🞏** | **🞏** | **🞏** | **🞏** | **🞏** |
| The injection regimen makes me happier with my appearance | **🞏** | **🞏** | **🞏** | **🞏** | **🞏** |
| I am no longer concerned about my sagging skin on my cheeks | **🞏** | **🞏** | **🞏** | **🞏** | **🞏** |
| The results make me feel more attractive | **🞏** | **🞏** | **🞏** | **🞏** | **🞏** |
| This is the best that my face has looked since my weight loss | **🞏** | **🞏** | **🞏** | **🞏** | **🞏** |

| *Preference questions* | Yes | No | No Difference |
| --- | --- | --- | --- |
| I would recommend this injection regimen to others after weight loss | **🞏** | **🞏** | **🞏** |
| I would recommend this injection regimen to those with loose, sagging facial skin | **🞏** | **🞏** | **🞏** |
| I would continue getting this injection regimen to maintain my look | **🞏** | **🞏** | **🞏** |

**Testimonials** (please provide any comments on your experience, study products, satisfaction / dissatisfaction, or anything related to this study)

___________________________________________________________________________________

___________________________________________________________________________________

___________________________________________________________________________________

___________________________________________________________________________________

___________________________________________________________________________________
