# Supplementary figures and images for: A Multicenter, Open-Label Study of Combined Poly-L-Lactic Acid and Hyaluronic Midface Filler Regimen Enhances Facial Harmony and Skin Quality in GLP-1 Medication Users
Source: Aesthet Surg J. 2025 Nov 17;46(5):509–19. doi: 10.1093/asj/sjaf240 (PMC13064655; doi:10.1093/asj/sjaf240)

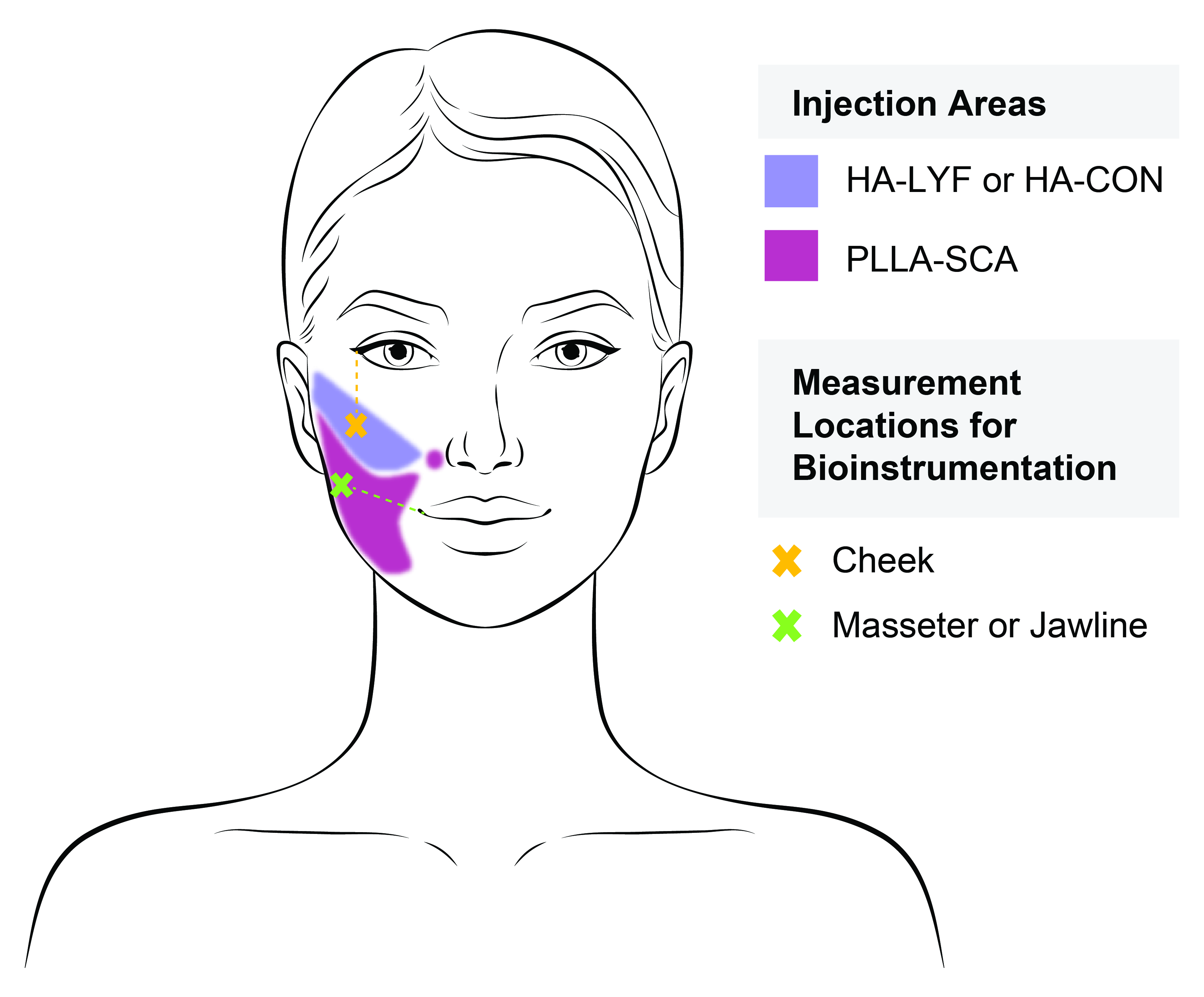

Supplement: sjaf240_Supplementary_Data [file sjaf240_supplementary_data.zip › Supp Fig 1.jpg]

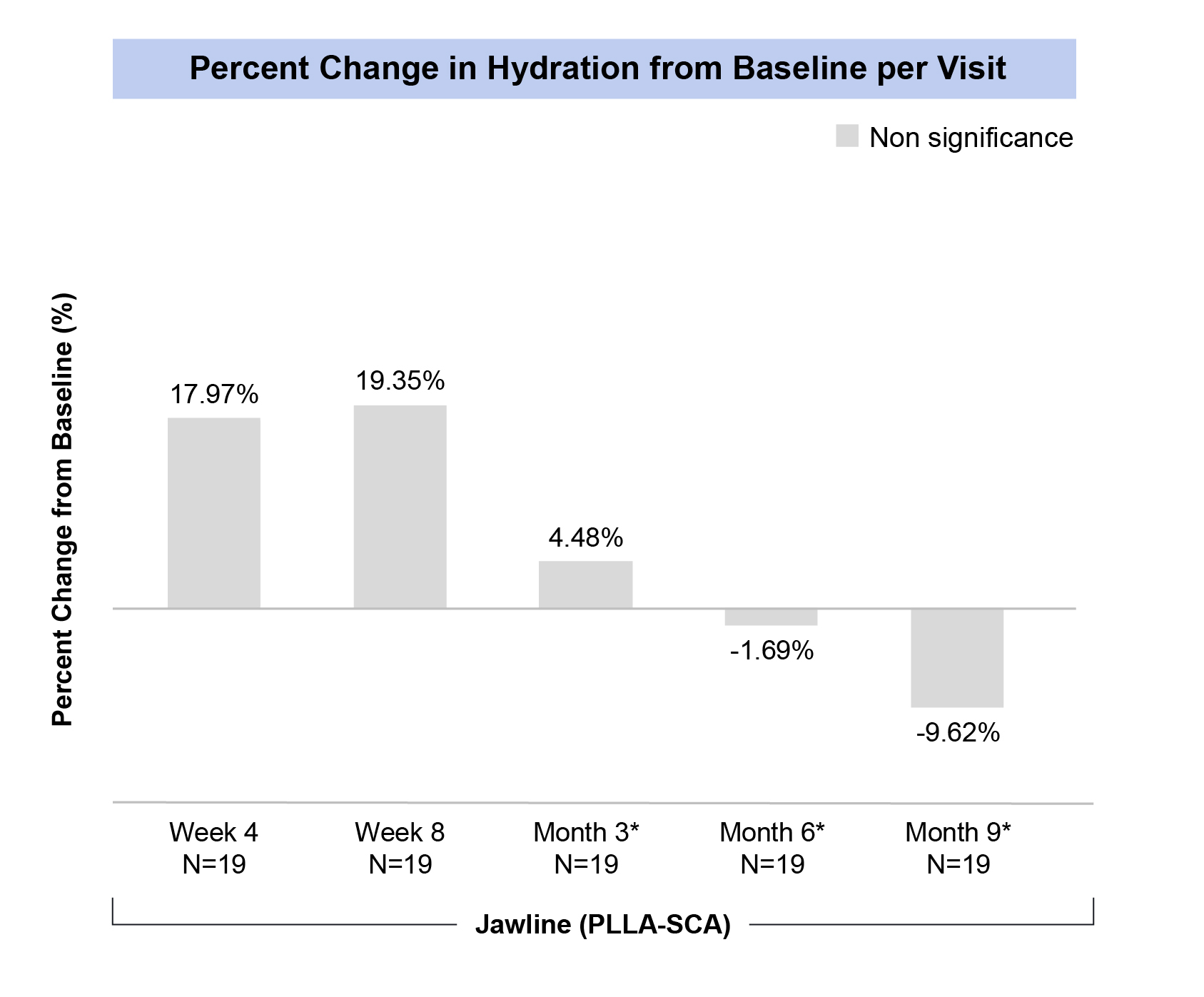

Supplement: sjaf240_Supplementary_Data [file sjaf240_supplementary_data.zip › Supp Fig 2.jpg]

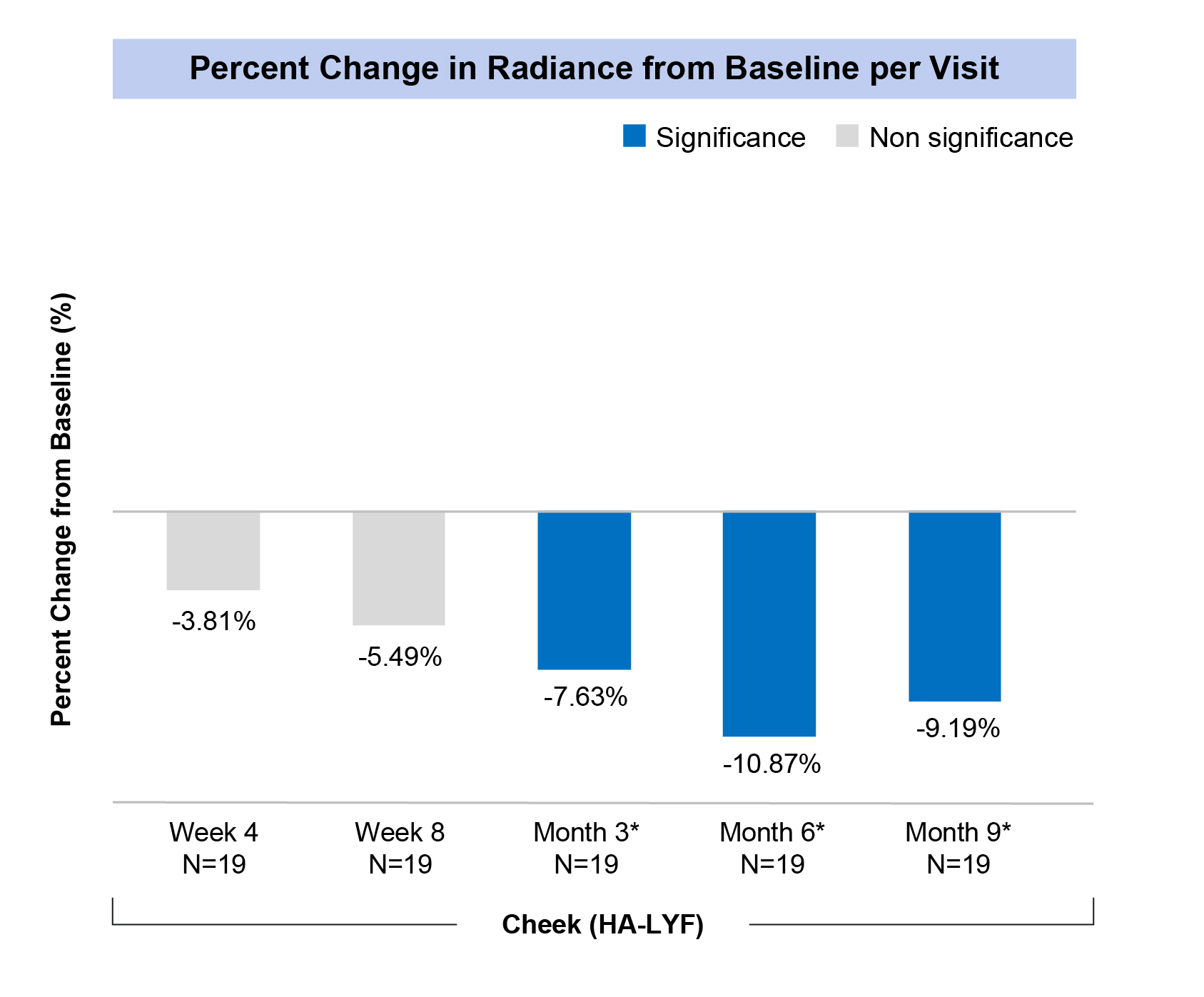

Supplement: sjaf240_Supplementary_Data [file sjaf240_supplementary_data.zip › Supp Fig 3.jpg]

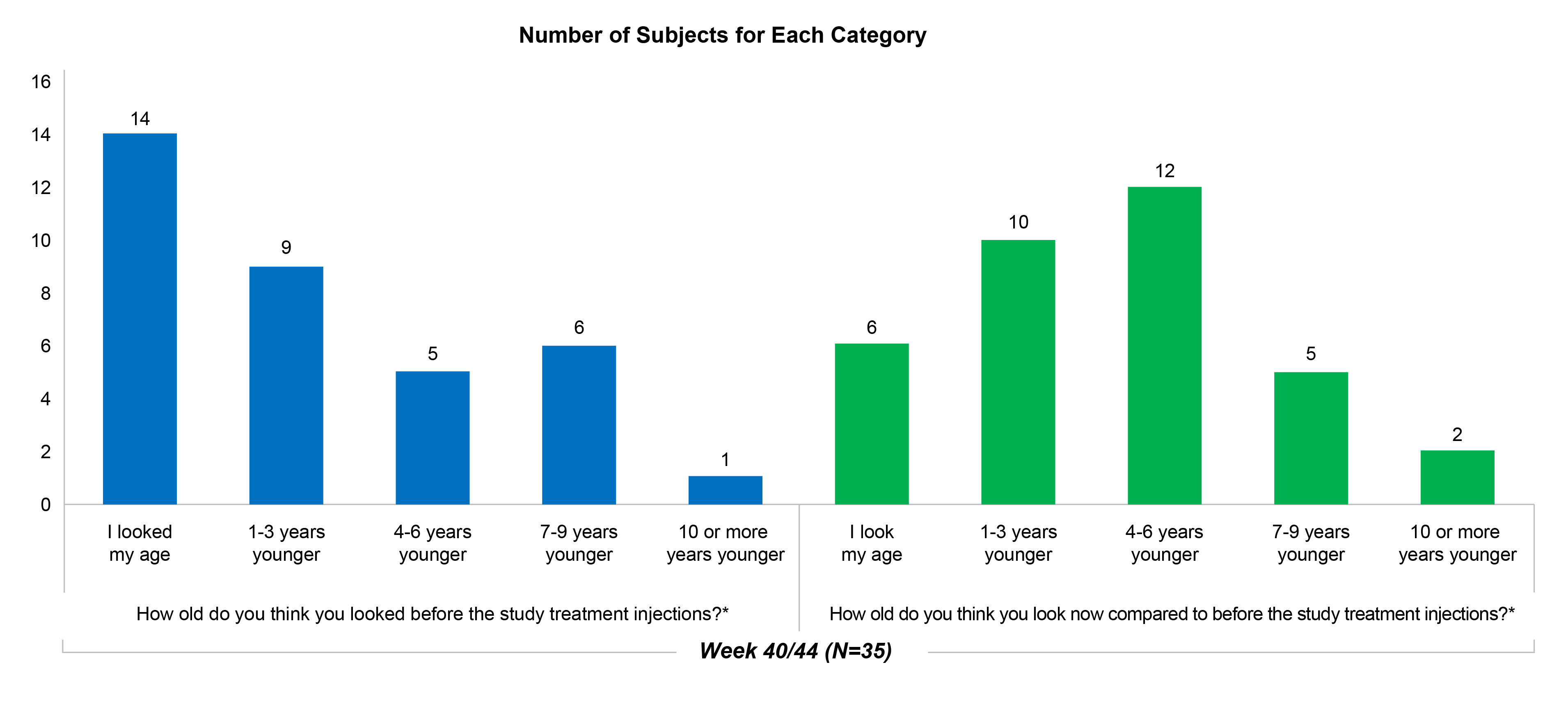

Supplement: sjaf240_Supplementary_Data [file sjaf240_supplementary_data.zip › Supp Fig 4.jpg]
